# Hospital-Based Mortality in Federal Capital Territory Hospitals-Nigeria, 2005 - 2008

**Published:** 2012-04-11

**Authors:** Nykiconia Preacely, Oladayo Biya, Saheed Gidado, Halima Ayanleke, Mohammed Kida, Moses Akhimien, Aisha Abubakar, Ibrahim Kurmi, Ikeoluwapo Ajayi, Patrick Nguku, Henry Akpan

**Affiliations:** 1 US Centers for Disease Control and Prevention, Epidemic Intelligence Service, Office of Surveillance, Epidemiology, and Laboratory Services, and Division of Public Health Systems and Workforce Development, Center for Global Health Atlanta, GA, USA; 2 Nigeria Field Epidemiology and Laboratory Training Program, Abuja, Nigeria; 3 University of Ibadan, Ibadan, Nigeria; 4 Nigeria Federal Ministry of Health, Abuja, Nigeria

**Keywords:** Hospital, mortality, surveillance, causes of death, Nigeria

## Abstract

**Background:**

Cause-specific mortality data are important to monitor trends in mortality over time. Medical records provide reliable documentation of the causes of deaths occurring in hospitals. This study describes all causes of mortality reported at hospitals in the Federal Capital Territory (FCT) of Nigeria.

**Methods:**

Deaths reported in 15 secondary and tertiary FCT hospitals occurring from January 1, 2005 and December 31, 2008 were identified by a retrospective review of hospital records conducted by the Nigeria Field Epidemiology and Laboratory Program (NFELTP). Data extracted from the records included sociodemographics, geographic area of residence and underlying cause-of-death information.

**Results:**

A total of 4,623 deaths occurred in the hospitals. Overall, the top five causes of death reported were: HIV 951 (21%), road traffic accidents 422 (9%), malaria 264 (6%), septicemia 206 (5%), and hypertension 194 (4%). The median age at death was 30 years (range: 0-100); 888 (20%) of deaths were among those less than one year of age. Among children < 1 year, low birth weight and infections were responsible for the highest proportion 131 (15%) of reported mortality.

**Conclusion:**

Many of the leading causes of mortality identified in this study are preventable. Infant mortality is a large public health problem in FCT hospitals. Although these findings are not representative of all FCT deaths, they may be used to quantify mortality in that occurs in FCT hospitals. These data combined with other mortality surveillance data can provide evidence to inform policy on public health strategies and interventions for the FCT.

## Background

Cause-specific mortality data are important to monitor trends in mortality over time. Nigeria is one of several World Health Organization Regional Office for Africa (WHO-Afro) countries with no recent data available on adult mortality [1]). Although Nigeria decreed the registration of all vital events in 1979, the country has yet to implement compulsory registration. Because most deaths in Nigeria are not registered it is not possible to generate comprehensive population-based mortality data. Hospital-based data recorded by medically-qualified staff can yield useful information to characterize mortality which has occurred in hospitals. From 1994-2007 3,004 deaths were registered in the Federal Capital Territory (FCT) of Nigeria, 1,778 (60%) occurred in hospitals [2]. The FCT Health and Human Services Secretariat (HHSS) provides regulation and oversight for all public and private medical and hospital services in the FCT. The annual FCT Health Statistical Bulletin which contains statistical information on health activities carried out by HHSS lacks descriptive data on mortality occurring in hospitals. The existing hospital mortality data reporting system in the FCT includes reports of mortality frequency by gender distribution only [3].

FCT is home to Abuja, the nation's capital; it is divided into six area councils with a 2008 estimated population of 1,404,201 people and covers an area of 2,824.3 sq. miles. In September 2009, a retrospective review of hospital-based mortality data in the FCT was conducted by the Nigeria Field Epidemiology and Laboratory Program (NFELTP) to identify sociodemographic characteristics and leading underlying causes of mortality among patients admitted to FCT hospitals. This report describes the results of that study, which found almost 40% of deaths among children aged less than 1 year were related to adverse pregnancy outcomes including prematurity, low birth weight, and jaundice. Malaria was the leading cause of death reported among children and adolescents aged 1-14 and HIV was the leading cause of death among adults aged 15-64 years. Data from this study combined with community-based mortality data can be used to identify risk factors and set priorities for public health interventions.

## Methods

NFELTP reviewed medical records of patients seen at all secondary, tertiary, and major private hospitals in the FCT between January 1, 2005 and December 31, 2008. A total of 15 hospitals were included: 2 tertiary government hospitals, 11 secondary government hospitals, and 2 high capacity private hospitals selected purposively, based on annual number of patients treated. Of the selected hospitals, one did not have data available for 2005 and one only had available data for 2008. Data extracted from the records included date of birth, gender, occupation, date of death, geographic area of residence, date of hospital admission, and underlying cause of death (COD) as listed in the medical record was categorized using the International Classification of Disease, Tenth Revision. Age at time of death was calculated by subtracting date of death from date of birth. Length of stay (LOS) was calculated by subtracting date of death from date of hospital admission. For this report, COD frequency and percentage was classified by leading cause group and major cause subcategories, age at time of death was grouped by year, and occupation reported as occupational group or employment status.

## Results

**Figure 1:**
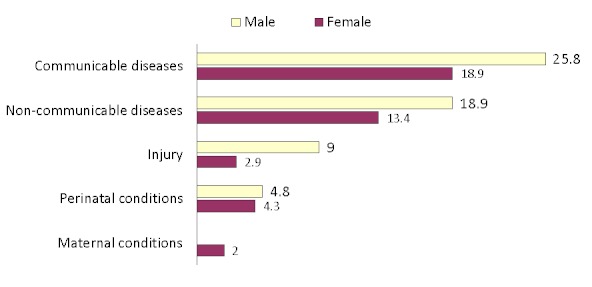
Percent distribution of total deaths by leading cause group and sex, Federal Capital Territory (FCT), Nigeria Hospitals, 2005-2008

During January 1, 2005-December 31, 2008, a total of 4,623 deaths were reported (Table 1). The average LOS was 5 days (median: 2 days; range: 0-181 days). Age at time of death was missing for 193 individuals, the average age at death for those with an age reported was 30 years (range: 0-100 years). Mortality occurred most frequently among those aged < 1 year (19.2%). The most commonly reported occupational group among those employed at the time of death was civil servant (14.9%). Majority of the decedents lived in the FCT and slightly over half of them (50.6%) resided in the Abuja Metropolitan Area Council (AMAC) at their time of death. Overall, most deaths were attributable to communicable diseases (Figure 1).

**Table 1:** Number and percentage of decedents by selected characteristics, Federal Capital Territory (FCT), Nigeria Hospitals, a, b 2005-2008

Characteristic	No.	(%)
**Sex**		****
Male	2724	(58.9)
Female	1883	(40.7)
Unknown	16	(0.4)
Total	4623	100%
**Age Group (years)**	****	****
<1	888	(19.2)
1-4	403	(8.7)
5-14	176	(3.8)
15-24	245	(5.3)
25-34	860	(18.6)
35-44	740	(16.0)
45-54	540	(11.7)
55-64	302	(6.5)
≥65	276	(6.0)
Unknown	193	(4.2)
Total	4623	100%
**Occupational Group/Employment Status**	****	****
Business & financial operations	435	(9.4)
Civil Servant	690	(14.9)
Housekeeping	464	(10.0)
Farming	109	(2.4)
Sales/Trading	140	(3.0)
Transportation/Professional Driver	96	(2.1)
Other specified group	550	(11.9)
Retired	92	(2.0)
Unemployed	114	(2.5)
Not employed (Child/Student)	1678	(36.3)
Unknown	255	(5.5)
Total	4623	100%
**FCT Area Council of Residence**	****	****
Abaji	68	(1.5)
Abuja Metropolitan	2341	(50.6)
Bwari	6	(0.1)
Gwagwalada	155	(3.4)
Kuje	97	(2.1)
Kwali	235	(5.1)
Outside FCT	1721	(37.2)
Total	4623	100%

a Abuja Clinics, Abaji General Hospital, Asokoro District Hospital, Bwari General Hospital, University of Abuja Teaching Hospital Gwagwalada, Gwarimpa General Hospital, Karshi General Hospital, Kubwa General Hospital, Kuje General Hospital, Kwali General Hospital, Maitama District Hospital, National Hospital, Nyanya General Hospital, Wuse General Hospital, Zankli Medical Center.

b No data available for Kuje General Hospital 2005, and Nyanya General Hospital for 2005-2007

Cause of death was missing for 2% of 888 children aged <1, 2% of 403 aged 1-4 years and 3% of 176 children aged 5-14. Among those aged <1 with a cause listed in the medical record, prematurity and low birth weight (15%) were reported most frequently, followed by sepsis (11%) and jaundice (10%). Malaria was the leading COD reported among children in the 1-4 (80 (20%) of 396) and 5-14 (41 (24%) of 171) year age groups. Pneumonia (11%) and sepsis (9%) were the second and third leading causes of death among children aged 1-4 while road traffic accidents (RTA) (13%) and HIV (9%) ranked as the second and third most common causes of death among 5-14 year olds.

Among adults aged =15 cause of death was not listed in the medical record for 4% (123) of deaths. HIV was the single leading cause of death reported collectively among adults aged =15 and the number one COD among adults in age groups 15-24 years (48 (21%) of 234), 25-34 years (307 (38%) of 819), 35-44 years (293 (41%) of 709), 45-54 years (151 (29%) of 522), and 55-64 years (40 (14%) of 295). However, HIV was not a leading COD among adults aged =65 years, accounting for only 1% (46 of 270) of deaths in this age group. Death due to injuries sustained in RTAs was the second overall leading COD among adults aged =15 years. RTAs also ranked as the second leading COD among young adults, aged 15-24 years and 25-34 years, and among middle aged adults 35-44 years (14%, 13%, and 9%, respectively). Hypertension, the third overall COD among adults aged =15 years was the second leading COD among those aged 45-54 years (11%) and 55-64 years (13%). Among adults aged =65 hypertension was the number one COD (17%).

**Figure 2:**
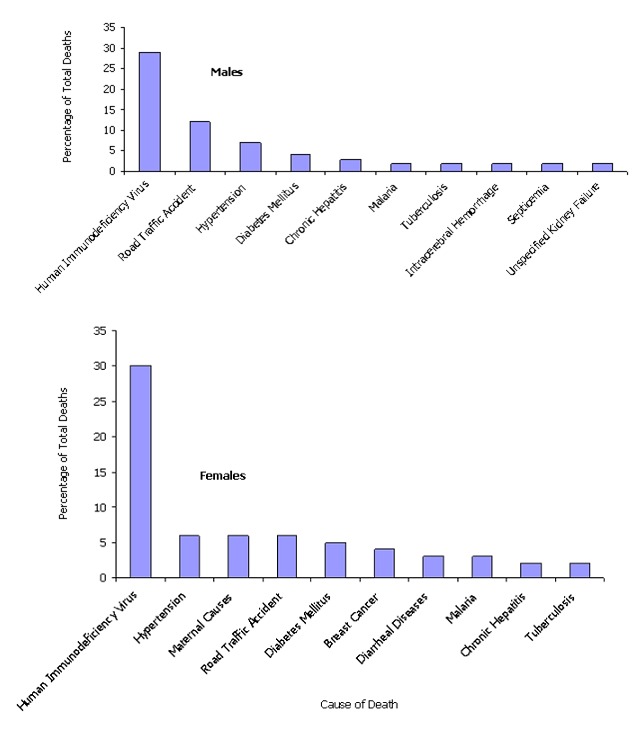
Percentage of total deaths by 10 leading causes for adults aged 15 and older, Federal Capital territory, Nigeria Hospitals, 2005-2008

Males and females shared many of the same 10 leading causes of death (Figure 2). The burden of death due to HIV was the leading cause of death reported among both sexes with 30% (355) of all female and 29% (480) of all male deaths due to this one cause. Among the top 10 causes of death, mortality related to maternal causes, breast cancer, and diarrheal diseases were reported solely among females as leading CODs while unspecified intracerebral hemorrhage, septicemia, and unspecified kidney failure were exclusive to the top 10 CODs in males (Figure 2).

Mortality occurred more frequently among women than in men for only two age groups, ages 15-24 and ages 25-34 (139 (59%) of 234 and 455 (56%) of 819 respectively). Although HIV was a leading cause of death for both females and males aged 15-24 and 25-34 years, death due to HIV occurred four times as often among females aged 15-24 years 39 (81%) and twice as often among those aged 25-34 years 202 (66%) when compared to males in the same age groups, 9 (19%) and 103 (34%) respectively. Maternal causes related specifically to pregnancy and childbirth, the second leading cause of death among females was associated with 10% of deaths for females in both age groups 15-24 years (14 of 139) and 25-34 years (44 of 455).

## Discussion

This report is the first to present descriptive data on patient characteristics related to cause-specific mortality in FCT hospitals. Many of the leading causes of death identified in this report are preventable. A substantial proportion of mortality among the <1 year age group was attributable to low birth weight newborns and infections. These findings are similar to findings of other hospital-based mortality studies conducted in Nigeria (4-5) and reflect multi-faceted maternal health problems such as lack of access to prenatal care, poor nutrition, malaria, or HIV/AIDS. High mortality due to malaria in children aged 1-14 years continues to be a problem in Nigeria evidenced by this and other studies (6). Although mortality from RTAs was the second leading COD among children 5-14 (13%) and adults aged =15 (9%), this frequency may be an underestimate of actual RTA mortality burden due to classification of death as “brought in dead” and injuries listed without an underlying cause. Similar to previous studies conducted in Nigeria (7-9), this report found HIV as a major COD among adults admitted to hospitals. However, these results may be underestimates due to lack of documentation of serologic test results. AMAC, being the administrative center of Nigeria, consists of mainly civil servants and their families. This likely explains the high proportion of deaths among the civil service occupational group.

The findings of this report are subject to at least three limitations. First, sociodemographic factors such as financial status, educational attainment, transportation access, and ethnic/cultural beliefs influence hospital utilization and thereby limit the findings of this review to a subset of the FCT's population. Second, varying methods used to assign COD (certifying physician failing to report a cause of death, lack of autopsy reports, few laboratory confirmed diagnoses, and diagnosis of symptoms or physical conditions of the decedent rather than the COD) could have led to over or under estimation of cause-specific mortality. Finally, poor hospital record keeping practices resulted in missing or incomplete data in the medical charts key variables such as unknown age for 6% (193) and “brought in dead” or COD not listed for 7% (344) of the deceased patients.

## Conclusion

The absence of cause-specific mortality among individuals that utilize FCT hospitals was a weakness of the existing mortality reporting system. This retrospective review was useful because it was an inexpensive project quickly conducted by the NFELTP residents that resulted in immediate action to strengthen mortality surveillance in the FCT. As an ongoing service to the HHHS, NFELTP residents are developing a prospective hospital-based mortality surveillance system. The findings of this report highlight the importance of improving the health of mothers and children, safety on roads, and HIV prevention in the FCT. Reliable cause-specific mortality data from sources outside health facilities are scare in Nigeria. The current literature is limited to studies conducted in rural areas in pediatric and maternal populations (10-12). Data from this study, integrated with comprehensive community-based mortality data, may be utilized to describe patterns of mortality in the FCT and set priority focal areas for public health interventions.
